# The natural retinoprotectant chrysophanol attenuated photoreceptor cell apoptosis in an *N*-methyl-*N*-nitrosourea-induced mouse model of retinal degenaration

**DOI:** 10.1038/srep41086

**Published:** 2017-01-23

**Authors:** Fan-Li Lin, Cheng-Hui Lin, Jau-Der Ho, Jing-Lun Yen, Hung-Ming Chang, George C. Y. Chiou, Yu-Wen Cheng, George Hsiao

**Affiliations:** 1 Graduate Institute of Medical Sciences and Department of Pharmacology, School of Medicine, College of Medicine, Taipei Medical University, Taipei, Taiwan; 2 School of Pharmacy, College of Pharmacy, Taipei Medical University, Taipei, Taiwan; 3 Department of Ophthalmology, Taipei Medical University Hospital, Taipei, Taiwan; 4 Department of Anatomy, School of Medicine, College of Medicine, Taipei Medical University, Taipei, Taiwan; 5 Department of Neuroscience and Experimental Therapeutics and Institute of Ocular Pharmacology, College of Medicine, Texas A&M Health Science Center, College Station, TX, USA

## Abstract

Retinitis pigmentosa (RP) is an inherited photoreceptor-degenerative disease, and neuronal degeneration in RP is exacerbated by glial activation. Cassia seed (Jue-ming-zi) is a traditional herbal medicine commonly used to treat ocular diseases in Asia. In this report, we investigated the retina-protective effect of chrysophanol, an active component of Cassia seed, in an *N*-methyl-*N*-nitrosourea (MNU)-induced mouse model of RP. We determined that chrysophanol inhibited the functional and morphological features of MNU-induced retinal degeneration using scotopic electroretinography (ERG), optical coherence tomography (OCT), and immunohistochemistry analysis of R/G opsin and rhodopsin. Furthermore, TUNEL assays revealed that chrysophanol attenuated MNU-induced photoreceptor cell apoptosis and inhibited the expression of the apoptosis-associated proteins PARP, Bax, and caspase-3. In addition, chrysophanol ameliorated reactive gliosis, as demonstrated by a decrease in GFAP immunolabeling, and suppressed the activation of matrix metalloproteinase (MMP)-9-mediated gelatinolysis. *In vitro* studies indicated that chrysophanol inhibited lipopolysaccharide (LPS)-induced iNOS and COX-2 expression in the BV2 mouse microglia cell line and inhibited MMP-9 activation in primary microglia. Our results demonstrate that chrysophanol provided neuroprotective effects and inhibited glial activation, suggesting that chrysophanol might have therapeutic value for the treatment of human RP and other retinopathies.

Retinitis pigmentosa (RP) refers to a heterogeneous group of inherited retinal degenerative diseases that are characterized by progressive photoreceptor cell death. Genetic aberrations are directly implicated in the apoptosis of rod photoreceptors and the development of night blindness. Neurotrophic factor depletion and increases in oxidative stress following massive rod cell death can also induce apoptosis in cone photoreceptors, thereby promoting the loss of bright and color vision. Modes of glial activation, including microglia recruitment and reactive gliosis, are also involved in the pathogenesis of RP in humans and in mouse models^1^,^2^,^3^. A recent study demonstrated that microglia infiltrate the outer nuclear layer during early stages of retinal degeneration^3^. In addition to removing debris and apoptotic neurons, dysfunctional microglia execute neuronal cell death via a process of phagoptosis^4^,^5^. RP commonly results in vision loss, especially in patients who develop RP in adolescence or infancy. Therefore, visual acuity is disrupted during the early stages of RP, resulting in significantly higher healthcare costs and disability-adjusted life years for these patients^6^. Although RP affects millions of individuals worldwide, there are no therapies currently approved for the treatment of this disease.

Cassia seed (Jue-ming-zi, *Cassia toro*) is a traditional herbal medicine widely used in Asia to treat eye complications^7^,^8^. Previous studies have demonstrated that Cassia seed provides clinical benefits to patients with ophthalmic conditions, such as glaucoma and cataracts^9^,^10^. Chrysophanol, an active anthraquinone isolated from Cassia seed, exhibits various pharmacological effects, including anti-oxidative^11^, anti-hyperlipidemia^12^, and anti-inflammatory effects^13^,^14^. However, few studies have evaluated the protective effects of chrysophanol in the context of ocular diseases. In the present study, we investigated the retina-protective effects of chrysophanol in an *N*-methyl-*N*-nitrosourea (MNU)-induced mouse model of RP *in vivo*^15^. Similar to the mechanism of cell death in human RP, MNU selectively induces cell death in photoreceptor cells via apoptosis^16^. Therefore, the MNU-induced mouse model of RP is a reliable and appropriate approach to investigate the neuroprotective effects and mechanisms mediated by bioactive compounds, such as chrysophanol, in RP.

Chrysophanol prevented the functional and morphological features of MNU-induced photoreceptor apoptosis and retinal degeneration, and ameliorated glial activation. These findings suggest that the primary active component of Cassia seed, chrysophanol, has the potential to inhibit RP progression and prevent retinal injury. However, the mechanisms underlying this phenomenon, as well as the toxicity and long-term effects of chrysophanol, require further investigation.

## Results

### *N*-methyl-*N*-nitrosourea (MNU)-induced photoreceptor cell loss and retinal dysfunction in a dose-dependent manner

**Figure 1:**
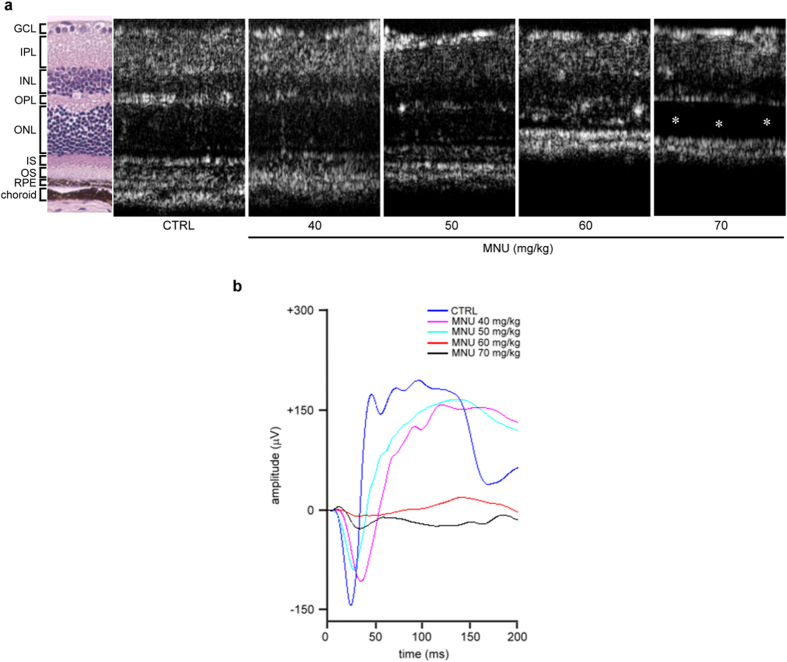
Morphological and functional defects in mouse retina 7 days after treatment with various doses of MNU. (**a**) OCT images revealed retinal ONL degeneration 7 days after the mice were treated with various doses of MNU. Retinal detachment (asterisk) was observed in the group treated with MNU (70 mg/kg). (**b**) Scotopic electroretinogram (ERG) responses of a- and b-waves elicited by light at an intensity of 19.1 cd · s/m^2^ were recorded at the same time point. CTRL: control; GCL: ganglion cell layer; IPL: inner plexiform layer; INL: inner nuclear layer; OPL: outer plexiform layer; ONL: outer nuclear layer; IS: inner segment; OS: outer segment; RPE: retinal pigment epithelium.

As shown in Fig. 1a, OCT images revealed that MNU-induced degeneration of the outer nuclear layer (ONL) in the retinas of C57BL/6 mice in a dose-dependent manner 7 days after it was administered. At day 7, photoreceptor degeneration resulting from photoreceptor cell loss was clearly observed in the MNU-treated group (60 mg/kg), and the total retinal thickness was significantly reduced. In addition, scotopic electroretinogram (ERG) responses of the a-wave and b-wave amplitude were strongly reduced in the MNU-treated group (60 mg/kg) compared to control mice (Fig. 1b). Consistently, delayed implicit time (time to peak) of a-wave and b-wave was found in MNU-treated group. These findings suggested that a significant loss in retinal function had occurred. Retinal detachment was observed in the 70 mg/kg MNU-treated group compared with the 60 mg/kg MNU-treated group. Therefore, MNU was used at a dose of 60 mg/kg in subsequent experiments.

### Chrysophanol treatment restored retinal function of elctroretinogram (ERG) in MNU-exposed photoreceptors

**Figure 2:**
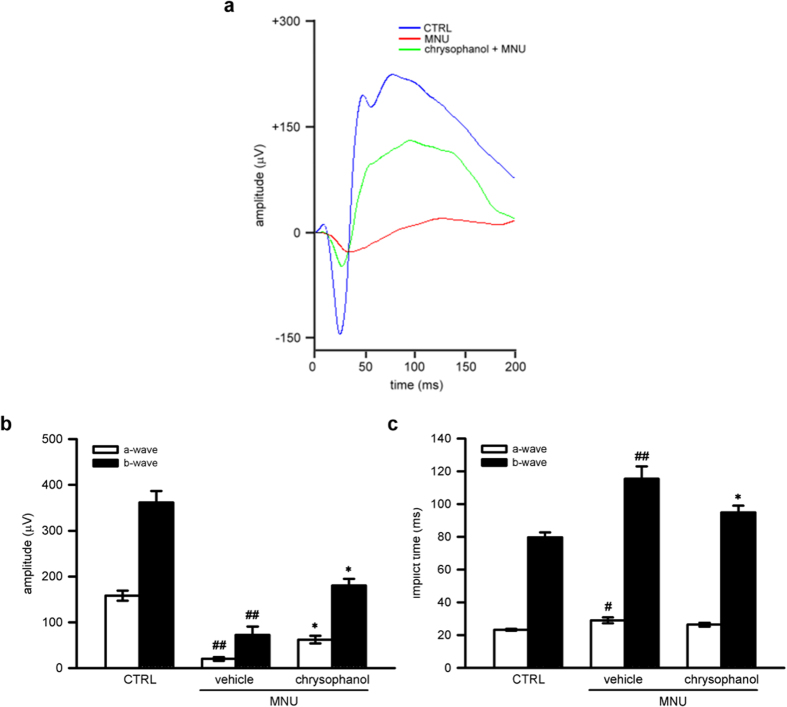
Chrysophanol preserved MNU-injured photoreceptor function on day 7. (**a**) Representative scotopic ERG responses from control mice (blue curve), and MNU-exposed mice treated with the vehicle control (red curve) or chrysophanol (green curve). (**b**,**c**) Quantification of the average amplitudes and implicit time (time to peak) from control mice (n = 7), and MNU-exposed mice treated with the vehicle control (n = 5) or chrysophanol (n = 11). The ERG a-waves were negative, and the a-wave amplitudes are presented as the absolute value. The data were presented as the mean ± SEM. CTRL: control. ^#^*p* < 0.05 compared with the control group treated with vehicle, ^##^*p* < 0.001 compared with the control group treated with vehicle, *p < 0.01 compared with MNU-exposed group treated with vehicle.

To assess whether chrysophanol treatment can restore photoreceptor cell function, we measured scotopic responses of electroretinogram (ERG) in MNU-exposed retinas. A standard scotopic ERG is composed of an a-wave and a b-wave^17^. The a-wave reflects the integrity of the combined rod and cone response, and the b-wave reflects the response of ON bipolar cells and the function of Muller cells^18^. On day 7, the a-wave amplitude and b-wave amplitude were strongly reduced in MNU-exposed retinas (60 mg/kg; a-wave, 20.0 ± 4.0 μV; b-wave, 72.3 ± 18.3 μV) compared with the control group (a-wave, 158.2 ± 11.2 μV; b-wave, 361.3 ± 25.5 μV). Chrysophanol (50 mg/kg) treatment rescued MNU-induced retinal dysfunction and increased ERG responses (a-wave, 62.0 ± 8.2 μV; b-wave, 180.1 ± 14.7 μV) (Fig. 2a,b). Moreover, delayed implicit time was found in MNU-exposed group (a-wave, 29.0 ± 1.8 milliseconds (ms); b-wave, 115.5 ± 7.5 ms) compared with the control group (a-wave, 23.3 ± 0.6 ms; b-wave, 79.7 ± 3.0 ms). Chrysophanol treatment protected against MNU-induced retinal dysfunction and improved ERG responses (a-wave, 26.4 ± 1.2 ms; b-wave, 94.9 ± 4.2 ms) (Fig. 2a,c).

### Chrysophanol inhibited MNU-induced photoreceptor degeneration

**Figure 3:**
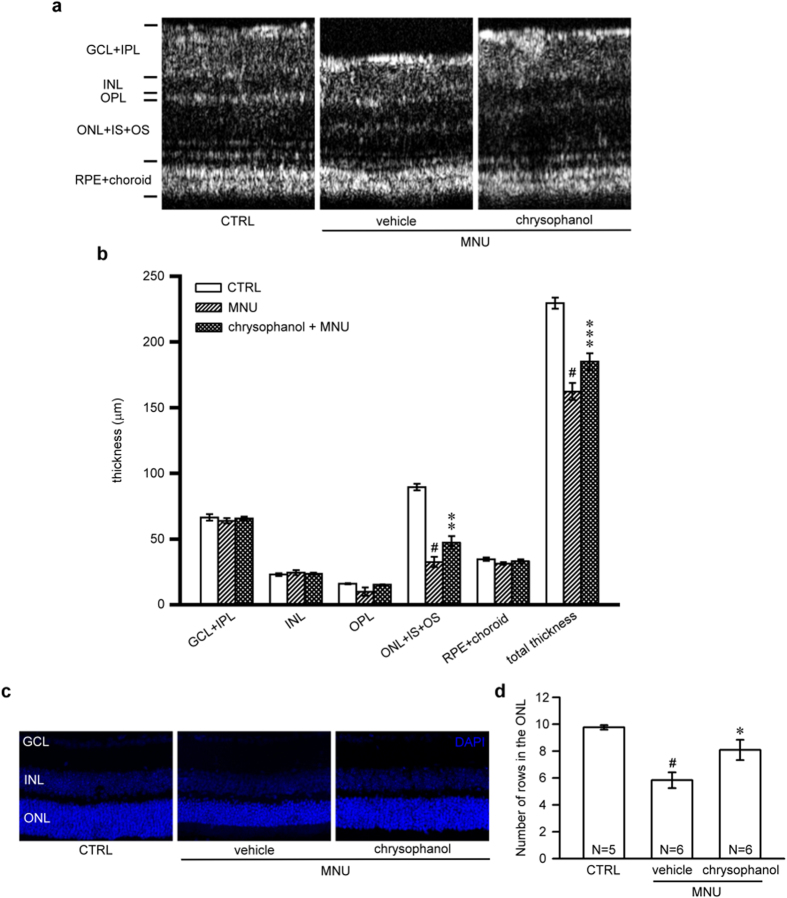
Chrysophanol attenuated MNU-induced photoreceptor degeneration on day 7. (**a**) OCT was used to evaluate changes in retinal thickness in control mice (n = 7) and in mice treated with the vehicle control (n = 6) or chrysophanol (n = 8) 7 days after the administration of MNU (60 mg/kg). (**b**) Quantification of the thickness of the separated layer using InSight XL software. (**c**) The arrangement of nuclei in the GCL, INL and ONL in DAPI-counterstained retina sections. (**d**) Quantification of the number of photoreceptor nuclei rows in the ONL. The data are presented as the mean ± SEM. CTRL: control; N: number of animals in each group; GCL: ganglion cell layer; IPL: inner plexiform layer; INL: inner nuclear layer; OPL: outer plexiform layer; ONL: outer nuclear layer; IS: inner segment; OS: outer segment; RPE: retinal pigmented epithelium. ^#^*p* < 0.001 compared with the control group treated with vehicle, *p < 0.05 compared with the MNU-exposed group treated with vehicle, **p < 0.01 compared with the MNU-exposed group treated with vehicle, ***p < 0.001 compared with the MNU-treated group treated with vehicle.

Representative OCT images of control retinas and MNU-exposed retinas treated with chrysophanol (50 mg/kg) or the vehicle control were shown in Fig. 3a. OCT images revealed that the cumulative thickness of the ONL, the inner segments (IS), and the outer segments (OS) layer (32.6 ± 3.9 μm) was significantly reduced 7 days after the administration of MNU compared with the control retinas (89.5 ± 2.5 μm). This effect was prevented by chrysophanol treatment (47.3 ± 5.0 μm). Consistent with these findings, the total thickness of MNU-exposed retinas (162.3 ± 6.5 μm) significantly decreased 7 days after the administration of MNU compared with the control group (229.5 ± 4.2 μm). This effect was significantly protected in retinas treated with chrysophanol (184.9 ± 5.4 μm) (Fig. 3a,b). DAPI staining demonstrated that the normal morphology of control retinas was characterized by three layers: the ganglion cell layer (GCL), the inner nuclear layer (INL), and the ONL (Fig. 3c). Compared with the control group, the number of rows of photoreceptor nuclei in the ONL was significantly reduced in MNU-exposed retinas (5.8 ± 0.6) compared to control group (9.8 ± 0.2), and this reduction was markedly prevented by chrysophanol treatment (8.1 ± 0.8; Fig. 3c,d). DAPI staining of photoreceptor nuclei demonstrated similar results acquired from OCT.

**Figure 4:**
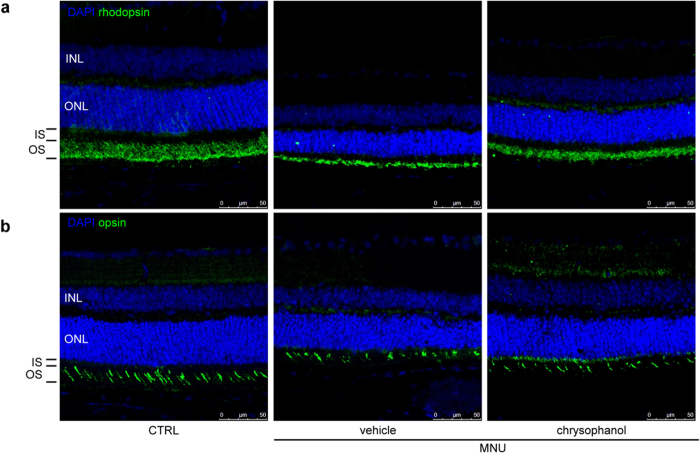
Chrysophanol rescued MNU-induced cone and rod degeneration on day 7. (**a**) The rod photoreceptors (labeled by rhodopsin in green) primarily localized to the OS. (**b**) The cone photoreceptors (labeled by red/green opsin in green) primarily localized to the OS. CTRL: control; INL: inner nuclear layer; ONL: outer nuclear layer; IS: inner segment; OS: outer segment.

To further evaluate the protective effect of chrysophanol on photoreceptor cell loss, we stained vertical retinal sections with antibodies against rhodopsin and red/green opsin to selectively label rod and cone photoreceptors, respectively^19^. The rod OS in MNU-treated retinas exhibited an abnormal, truncated pattern compared with control retinas (Fig. 4a), and chrysophanol treatment rescued the defects in OS organization and length. In addition to the protection of rods, we observed that chrysophanol inhibited MNU-induced damage to cone photoreceptors (Fig. 4b). Opsin immunostaining revealed that the amount and morphology of the MNU-exposed cone OS was disrupted compared with the well-arranged pattern observed in the control group, and this defect was rescued by chrysophanol treatment. Therefore, chrysophanol protected both rod and cone cells from MNU-induced morphological changes.

### Chrysophanol inhibited MNU-induced photoreceptor cell apoptosis and the expression of apoptosis-associated proteins

**Figure 5:**
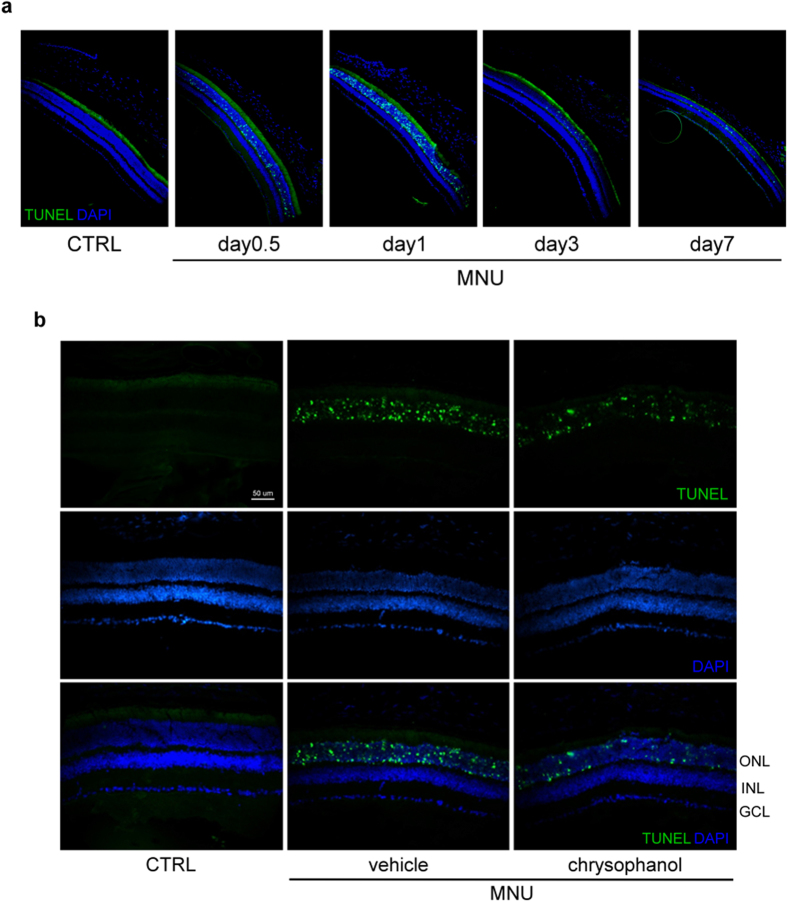
Chrysophanol significantly reduced MNU-induced photoreceptor cell death on day 1. (**a**) The time course of retinal cell death after MNU (60 mg/kg) administration as determined by TUNEL-labeling (green dot). The number of apoptotic cells peaked 1 day after the administration of MNU. (**b**) TUNEL-stained sections revealing dead photoreceptors in DAPI-counterstained retinal sections. CTRL: control; GCL: ganglion cell layer; INL: inner nuclear layer; ONL: outer nuclear layer.

The signals of TUNEL were found in the ONL after 12 hours exposure of MNU (60 mg/kg). Peak levels of apoptosis were observed on day 1, and the number of apoptotic cells gradually decreased on days 3 and 7 (Fig. 5a). Chrysophanol treatment significantly reduced the number of TUNEL-labeled cells in the ONL (Fig. 5b) 1 day after MNU-exposure. These results suggested that chrysophanol treatment blocked early photoreceptor cell apoptosis in MNU-exposed retinas.

**Figure 6:**
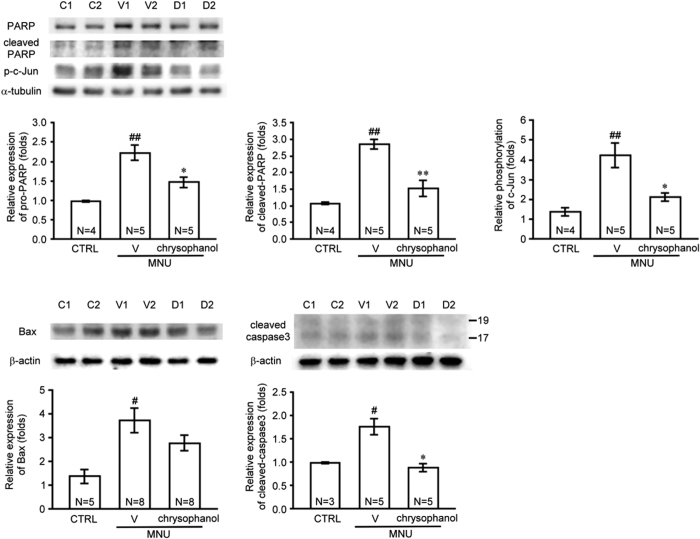
Chrysophanol inhibited MNU-induced expression of apoptosis-related proteins. PARP, Bax, caspase-3, and p-c-Jun protein levels in retinal homogenates were evaluated using western blot. All the Western blotting experiments were performed under the same conditions. After transferring the blots onto nitrocellulose membranes, we immediately cropped the targeted blots according to referenced indicating markers (Supplementary Fig. S1), and then targeted proteins were immunoblotted with its specific monoclonal antibody. The quantification values were presented as the mean ± SEM of at least 3 retinas from each group. Representative bands from 2 independent experiments for each group: the control (C1, C2), vehicle-treated (V1, V2), and chrysophanol-treated (D1, D2) groups. C, CTRL: control; V: vehicle; D: chrysophanol-treated; N: number of animals in each group. ^#^*p* < 0.01 compared with the control groups treated with vehicle, ^##^*p* < 0.001 compared with the control groups treated with vehicle, *p < 0.01 compared with the MNU-treated group treated with vehicle, **p < 0.001 compared with the MNU-treated group treated with vehicle.

To identify the mechanism underlying the anti-apoptotic effect of chrysophanol, we examined the expression of PARP, caspase-3, Bax, and phosphorylated c-Jun in MNU-exposed retinas (Fig. 6). Pro-PARP and cleaved-PARP levels increased 2.3- and 2.7-fold, respectively, in MNU-exposed retinas compared with control retinas. And pro-PARP and cleaved-PARP levels significantly decreased 1.5- and 1.9-fold in the chrysophanol-treated MNU-exposed group compared with the MNU-exposed group, respectively. In addition, Bax levels were 2.7-fold higher in MNU-exposed retinas compared with control retinas, whereas Bax levels decreased 1.3-fold in the chrysophanol-treated MNU-exposed retinas compared with MNU-exposed retinas. Compared with control retinas, caspase-3 levels increased 1.8-fold in MNU-exposed retinas, and chrysophanol significantly reduced caspase-3 levels by 1.9-fold compared with the MNU-exposed retinas. In addition, phosphorylated c-Jun levels increased 3.1-fold in MNU-exposed retinas compared with control retinas and decreased 1.9-fold in the chrysophanol-treated group compared with the MNU-exposed group. Together, these results revealed that the anti-apoptotic effect of chrysophanol on photoreceptor cells exposed to MNU was mediated by the down-regulation of PARP, Bax, caspase-3, and phosphorylated c-Jun levels.

### Chrysophanol inhibited glial fibrillary acidic protein (GFAP) expression and MMP-9 activation in MNU-exposed retinas

**Figure 7:**
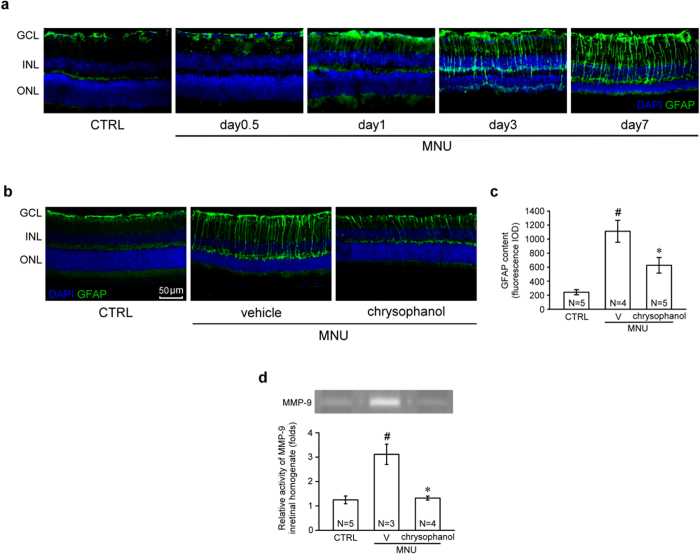
Chrysophanol inhibited MNU-induced reactive gliosis and retinal MMP-9 activation on day 7. (**a**) The time course of GFAP localization (green) after MNU (60 mg/kg) administration. (**b**) GFAP content in DAPI-counterstained retinal sections. (**c**) MMP-9 gelatinolytic activity in retinal homogenates was detected using zymography. All the zymographic experiments were performed under the same conditions. After gelatin staining, we immediately cropped the gelatinolytic regions in the gels (Supplementary Fig. S2). The data are presented as the mean ± SEM. CTRL: control; V: vehicle; N: number of animals in each group; GCL: ganglion cell layer; INL: inner nuclear layer; ONL: outer nuclear layer; IOD: integrated optical density. ^#^*p* < 0.001 compared with the control group treated with vehicle, *p < 0.001 compared with the MNU-exposed group treated with vehicle.

In the resting phase, GFAP localization is primarily restricted to the nerve fiber layer (NFL) and the GCL. Immunofluorescence assays demonstrated that GFAP was progressively up-regulated in a time-dependent manner in mice exposed to MNU (60 mg/kg) (Fig. 7a). These GFAP-positive processes were twisted and extended from GCL to INL. GFAP protein levels strongly increased and extended in MNU-treated group on day 7, and this effect was significantly inhibited by chrysophanol (Fig. 7b,c). The expression and activation of matrix metalloproteinase-9 (MMP-9) has previously been observed in retinopathies, and this effect is directly associated with an increase in GFAP expression^20^. Therefore, we evaluated MMP-9 activation in our MNU-induced mouse model of RP. The results of gelatin-zymography analysis demonstrated that MMP-9 expression and activation was significantly enhanced in MNU-exposed retinas (3.1 ± 0.4-fold) compared with control retinas (1.2 ± 0.2-fold; Fig. 7d). Chrysophanol (50 mg/kg) significantly inhibited MNU-induced gelatinolysis (1.3 ± 0.1-fold). These results suggested that MMP-9 activity is positively correlated with the pathological cascade of MNU-induced retinal injury. Furthermore, chrysophanol effectively inhibited MMP-9 activation *in vivo.*

### Chrysophanol concentration-dependently inhibited lipopolysaccharide (LPS)-induced expression of inducible nitric oxide synthase (iNOS) and cyclooxygenase-2 (COX-2) in BV-2 cells and inhibited MMP-9 gelatinolysis in primary microglia

**Figure 8:**
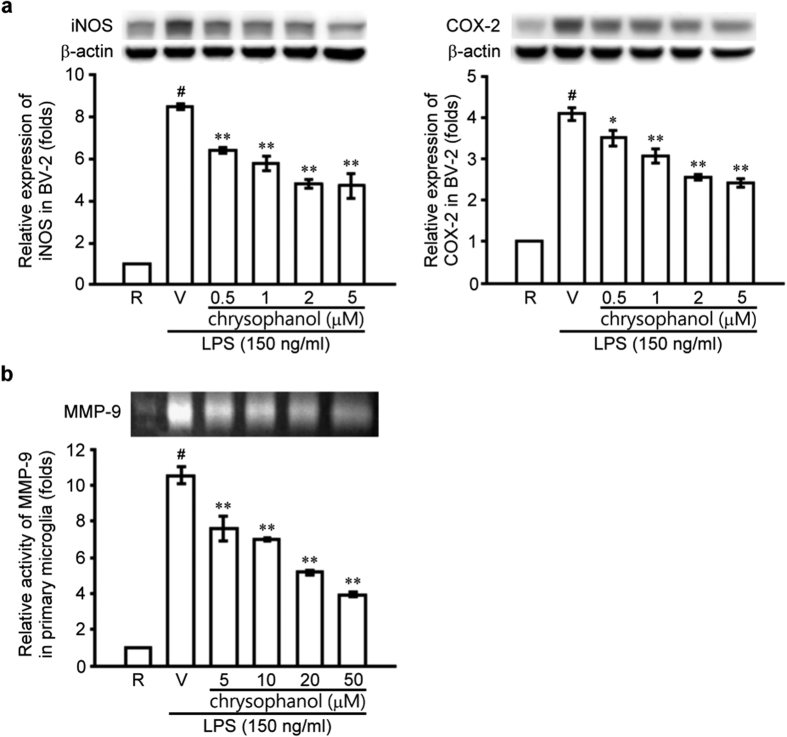
Chrysophanol inhibited iNOS and COX-2 expression and MMP-9 activation in activated microglia. (**a**) Microglial BV2 cells were pretreated with the vehicle (DMSO) or chrysophanol (0.5, 1, 2, and 5 μM) and stimulated with LPS (150 ng/ml) for 24 hours. iNOS and COX-2 expression in cell lysates was evaluated using western blot. All the Western blotting experiments were performed under the same conditions. After transferring the blots onto nitrocellulose membranes, we immediately cropped the targeted blots according to referenced indicating markers (Supplementary Fig. S3), and then targeted proteins were immunoblotted with its specific monoclonal antibody. (**b**) Primary microglia cells were pretreated with the vehicle (DMSO) or chrysophanol (5, 10, 20, and 50 μM) and stimulated with LPS (150 ng/mL) for 24 hours. MMP-9 activation in cell supernatants was evaluated using zymography. All the zymographic experiments were performed under the same conditions. After gelatin staining, we immediately cropped the gelatinolytic regions in the gels (Supplementary Fig. S3). The data are presented as the mean ± SEM of at least 3 experiments in each group. R: resting; V: vehicle. ^#^*p* < 0.001 compared with the control group treated with vehicle, *p < 0.01 compared with the LPS-exposed group treated with vehicle, **p < 0.001 compared with the LPS-exposed group treated with vehicle.

Microglial activation plays a key role in the host inflammatory response and contributes to the pathogenesis of retinal degeneration^21^,^22^,^23^. Accordingly, photoreceptor proteins released from RP retinas could activate microglia via Toll-like receptor 4 (TLR4) signaling^3^,^24^,^25^. Therefore, we used lipopolysaccharide (LPS), a TLR4 agonist, as an activator to stimulate the microglia cell line BV2. LPS (150 ng/mL) significantly increased iNOS and COX-2 protein levels in BV2 microglial cells (Fig. 8a), and chrysophanol (0.5–5 μM) significantly inhibited LPS-induced iNOS and COX-2 expression in a concentration-dependent manner. We also examined MMP-9 gelatinolytic activity in LPS-stimulated primary microglia (Fig. 8b) and found that MMP-9 activation was significantly induced and activated after LPS stimulation (up to 10.5 ± 0.5-fold) compared with the control group. In addition, chrysophanol significantly and concentration-dependently inhibited MMP-9 activation in primary microglia (Fig. 8b).

## Discussion

Among the retinal degenerative diseases, RP is hereditarily characterized by progressive rod and cone photoreceptor cell death. In fact, photoreceptor apoptosis is a commonly observed mode of cell death in ocular diseases such as RP and age-related macular disease (AMD)^26^. Therefore, blocking retinal apoptosis might delay the progression of RP or AMD^27^. MNU is a potent retinal toxin that promotes retinal degeneration^28^. Specifically, MNU induces the degeneration of the ONL, a region abundant in photoreceptor cells, and this effect resembles the pathogenesis of end-stage human RP^15^. The present study was conducted to investigate the potential anti-apoptotic and retinal-protective effects of chrysophanol in an MNU-induced mouse model of RP. Based on the previous studies that evaluated the therapeutic profiles of Cassia seed and chrysophanol in ophthalmic and neuroprotective studies^9^,^29^, chrysophanol was used at a dose of 50 mg/kg in this study. We demonstrated that chrysophanol treatment attenuated photoreceptor cell degeneration and improved scotopic ERG responses in MNU-exposed retinas. Notably, chrysophanol effectively restored the integrity and structure of both rod and cone photoreceptors 7 days after MNU-induced retinal injury. Moreover, we found that the protective effect of chrysophanol in MNU-exposed retinas was mediated by the inhibition of photoreceptor cell apoptosis, proliferative gliosis, microglial activation, and MMP-9 activation.

A recent study reported that apoptosis-associated DNA fragmentation was observed in rat retinas during 7 days after MNU exposure^30^. Consistent with these findings, we observed an increase in TUNEL-labeled apoptotic cells in the ONL of mouse retinas 1 day after MNU exposure, and chrysophanol significantly abrogated this effect. An increase in the expression of apoptotic effectors demonstrated that the DNA adduct formation caused by MNU induced poly ADP-ribose polymerase (PARP) activation, further inducing cell death by depleting cellular energy reserves^31^. PARP has been shown to regulate activator protein-1 (AP-1)^32^, a dimeric transcription factor primarily composed of c-Jun and c-Fos that plays an important role in the regulation of cell proliferation and apoptosis^33^. Previous studies demonstrated that the induction of c-Jun and the phosphorylation of c-Jun at its N-terminal are associated with neuronal apoptosis^34^,^35^. In the apoptosis cascade, Bax is up-regulated 24 hours after the administration of MNU, and cysteine aspartate-specific protease-3 (caspase-3) coordinates and executes the apoptotic pathway^36^,^37^. A previous study indicated that inhibiting both PARP activity and the JNK/AP-1 signaling pathway blocks MNU-induced photoreceptor cell apoptosis^30^. Consistent with these findings, we found that chrysophanol attenuated the MNU-induced increase in PARP, cleaved PARP, phosphorylated c-Jun, Bax and cleaved caspase-3 levels 1 day after the administration of MNU. In summary, our findings suggested that the anti-apoptotic effects of chrysophanol were mediated by the inhibition of PARP activation, c-Jun phosphorylation, and apoptotic effectors.

Nearly all retinal diseases are associated with Müller cell and astrocyte gliosis^38^,^39^. The various consequences of proliferative gliosis, including the blood-retinal barrier breakdown, antioxidant deficiency, monocyte recruitment, and aggravated neuronal cell death^19^,^40^. In the present study, we evaluated the inhibitory effects of chrysophanol on glial cell activation. Glial fibrillary acidic protein (GFAP), a protein that localizes to the end-feet and processes of astrocytes and Müller cells, is a biomarker of gliosis in retinal degeneration^38^,^41^. In the present study, chrysophanol significantly attenuated GFAP overexpression in MNU-exposed retinas.

An increase in matrix metalloproteinase-9 (MMP-9) activity and colocalization of MMP-9 and GFAP have been observed in other animal models of retinopathy, such as the optic nerve ligation-induced mouse model of retinopathy^20^. Furthermore, several studies have demonstrated that increased MMP-9 activity plays a critical role in neuronal cell death in retinopathies^42^,^43^,^44^. Therefore, we investigated MMP-9 activity in MNU-exposed retinas. As expected, MMP-9 expression and activity significantly increased in MNU-exposed retinas, and this phenomenon was inhibited by chrysophanol. Although the inhibitory effect of chrysophanol on MMP-9 activity was directly associated with an inhibition of photoreceptor cell apoptosis, a more detailed characterization of this correlation requires additional experiments.

Microglia activation is associated with photoreceptor degeneration and subsequent primary rod photoreceptor cell death in RP in humans and in mouse models^1^,^3^,^24^. A previous study found that microglia migrate to the outer retina and phagocytose rod cell debris^1^. Photoreceptor proteins released from RP retinas could activate microglia via Toll-like receptor 4 (TLR4) signaling, and TLR4-mediated microglial phagoptosis might enhance neuronal death by promoting the phagocytosis of injured but living photoreceptors^3^,^24^,^25^. Therefore, we used lipopolysaccharide (LPS), a TLR4 agonist, as an activator to stimulate the microglia cell line BV2. In addition, an increase in the expression of the proinflammatory effectors inducible nitric oxide synthase (iNOS) and cyclooxygenase-2 (COX-2), as well as other factors expressed in activated microglia, enhanced photoreceptor apoptosis^21^,^23^,^45^,^46^. A previous clinical study reported that the aqueous humor and levels of serum nitric oxide (NO) species are enhanced in RP patients compared with healthy controls, further suggesting that NO exacerbates oxidative damage to cone cells in RP^47^. In this study, we found that chrysophanol significantly inhibited LPS-induced iNOS and COX-2 expression in a concentration-dependent manner.

A previous study indicated that down-regulating MMP-9 activity could prevent the apoptotic cell death of retinal ganglion cells^48^. Therefore, we investigated the inhibitory effect of chrysophanol on MMP-9 activation in microglia. Consistent with previous findings, we observed that chrysophanol inhibited MMP-9 activation in LPS-activated primary microglia in a concentration-dependent manner. Taken together, these findings indicated that chrysophanol effectively inhibited microglia activation by down-regulating inflammatory factors and MMP-9 activity and that these effects potentially inhibited disease progression.

In conclusion, we demonstrated that chrysophanol prevented the functional and morphological features of MNU-induced retinal degeneration. The retina-protective effects provided by chrysophanol might be mediated by the inhibition of apoptosis, proliferative gliosis, microglia activation, and MMP-9 induction. We observed that chrysophanol provides neuroprotective effects on retinas, suggesting that chrysophanol might have therapeutic value for the treatment of RP and retinal injury. Further studies investigating the precise mechanisms underlying the protective and long-term effects associated with chrysophanol are warranted.

## Methods

### Animals and treatment

Male C57BL/6 mice (25–30 g body weight) were obtained from BioLASCO Taiwan Co., Ltd (Taipei, Taiwan) and maintained in a 12 hour light/12 hour dark cycle at 25 ± 1 °C and 39–45% relative humidity. Water and food were available *ad libitum*. All animal procedures were approved by the Institutional Animal Care and Use Committee of Taipei Medical University (LAC-2016-0159), and conducted in accordance with guidelines of ARVO statement for the use of animals in ophthalmic and vision research.

To characterize MNU-induced retinal degeneration, C57BL/6 mice were intraperitoneally injected with various doses of MNU (40, 50, 60, and 70 mg/kg; Chem Service, Westchester, PA, USA) and evaluated at day 1 or day 7 after the injection. The eyes were rapidly enucleated and processed as described below.

Chrysophanol (50 mg/kg; Sigma, St. Louis, MO) or the vehicle control (0.1% carboxymethyl cellulose) was orally administered using a feeding needle. For the 7 day experiments, chrysophanol was delivered daily for 3 days starting the day before MNU was administered. For the 1 day experiments, a single dose of chrysophanol was delivered prior to the administration of MNU.

### Scotopic electroretinography (ERG) analysis

Scotopic ERGs were used to evaluate the cone and rod photoreceptor responses to light stimulias previously described^49^. The electroretinography system was composed of a MP-36 4-channel amplifier and acquisition system (Biopac Systems, Inc., Pershore, UK) connected to a PS33-PLUS photic stimulator (Grass Technologies, Warwick, RI USA). Recordings were obtained using 10 msec flash stimuli with an intensity of 16 (19.1 cd · s/m^2^). ERG a-wave amplitudes were measured from baseline to the negative peak, and the b-wave amplitude was measured from the trough of the a-wave to the peak of the positive wave. The temporal properties of the ERG response were defined by the time-to-peak (implicit time) of the a- and b-wave, and are measured from stimulus onset to the peak of the a- and b-wave. OCT imaging was performed immediately after the ERG recording as described below.

### Spectral-Domain Optical Coherence Tomography (SD-OCT) imaging

SD-OCT was used to non-invasively acquire cross-sectional tomographic images of the retina. This approach allowed for the monitoring of retinal morphology in live experimental animals. The Micron III intraocular imaging system (Phoenix Research Labs, Pleasanton, CA), composed of an OCT engine and a scanning lens, was used as previously described^49^. The resulting OCT image was imported into the InSight XL software (Phoenix Research Laboratories) to measure the total retinal thickness as well as the thickness of the different retinal layers.

### Tissue homogenization

All of the animals were euthanized with an injection of a ketamine and xylazine mixture, and the eye balls were immediately enucleated. After removing the extraocular tissue, each eye ball was transferred into a 2 mL Precellys homogenization tube containing five 2.8-mm ceramic beads (Bertin Technologies, Rockville, Maryland) and immersed in 200 μl of homogenization buffer supplemented with 1× protease inhibitor (Roche, Mannheim, Germany). The samples were homogenized using a tissue homogenizer (Minilys®; Bertin Technologies, Rockville, Maryland) at 5,000 rpm for 30 seconds. The homogenized sample was centrifuged at 5,000 g for 5 min at 4 °C, and the resulting supernatants were incubated at 4 °C for 30 min. The samples were subsequently centrifuged at 12,000 g for 20 min at 4 °C and stored at −80 °C prior to additional experiments.

### Immunofluorescence and TUNEL assays

To obtain retinal sections, the mouse eyes were resected immediately following transcardial perfusion with 4% paraformaldehyde (PFA) and fixed in Davidson’s fluid at 4 °C overnight. Then, the eyes were dehydrated with 30% sucrose, frozen-embedded in OCT, and serially sectioned at a thickness of 13 μm using a cryostat (Cryotome-E; Thermo Shandon, Cheshire, UK). The sections were pre-treated with citrate-EDTA buffer (pH 6.2) for epitope retrieval and subsequently incubated in blocking solution comprised of 10% normal goat serum and 0.5% Triton X-100 in 0.1 M phosphate buffer (pH 7.4) for 1 hour. Then, the sections were incubated with anti-GFAP (1:100; ProSci Inc., Poway, CA), anti-rhodopsin (1:50; Abcam, Burlingame, CA), and anti-red/green opsin antibody (1:100; Chemicon, Temecula, CA) at 4 °C overnight. The sections were rinsed and subsequently incubated with the fluorescence-conjugated secondary antibody Dylight 488 (1:200; GeneTex, Irvine, CA) for 2 hours at room temperature. Then, the sections were rinsed and mounted on cover slips using HIGHDEF^®^ IHC fluoromount (Enzo Life Sciences, Farmingdale, NY) with 2 μM 4,6-Diamidino-2-phenylindole (DAPI; AAT Bioquest, Inc., Sunnyvale, CA). DAPI-stained retinal sections were used to calculate the number of rows of photoreceptor nuclei in the outer nuclear layer. Three sections nearing the central retinal area were counted and averaged in each group, and 3 fields were counted per section. Apoptosis was evaluated using the terminal deoxynucleotidyl transferase dUTP nick and labeling (TUNEL) assay as previously described^49^ with some modifications. The results of the TUNEL assays are presented as immunofluorescence images.

The retinal sections were evaluated using an Eclipse 80i fluorescent microscope (Nikon Instruments, Melville, NY) and a Leica TCS SP5 confocal microscope imaging system (Leica Microsystems, Wetzlar, Germany) equipped with argon and diode lasers. ImageJ software was used to quantify the number of photoreceptor nuclei rows in the ONL and the fluorescence content of GFAP.

### Cell culture

The mouse BV-2 microglia cell line was cultured in DMEM supplemented with penicillin (90 units/ml), streptomycin (90 μg/mL), l-glutamine (3.65 mM), HEPES (18 mM), NaHCO_3_ (23.57 mM), and 10% heat-inactivated fetal bovine serum (FBS) at 37 °C in a humidified atmosphere (95% O_2_, 5% CO_2_). The cell culture conditions and cell treatments have been previously described^50^.

### Isolation of primary microglia

The protocol used to isolate primary microglia was conducted as previously described with some modifications^51^. After the rats were perfused, the brain was removed and the meninges were resected. The fresh brain tissue was transferred into a dish with 5 mL of 0.25% trypsin (Gibco, Burlington, Canada) supplemented with 10 μg/mL deoxyribonuclease I (Roche, Indianapolis, IN) and minced roughly by using scissors. After an incubation period, the brain tissues were further minced with 10 mL of DMEM (Gibco, Grand Island, NY) supplemented with 10% FBS, and centrifuged at 1,000 rpm for 10 min. The tissue pellet was resuspended in 10% DMEM supplemented with FBS and incubated for the indicated period of time. Microglia were harvested from flasks of mixed glial cell cultures shaken for 2 hours, and the cells were collected and prepared for further experiments.

### Western blot analysis

Western blot was used to analyze PARP, caspase-3, Bax and p-c-Jun levels in retinal homogenates, and iNOS and COX-2 levels in cell lysates as previously described^52^. The quantitative densitometry analysis was conducted using BIO-PROFIL Bio-1D light analytical software (Vilber Lourmat, Marue La Vallee, France). Protein levels are presented as the relative intensity compared with the control. The ratio of the optical density of the protein product to the internal control (α-tubulin or β-actin) was obtained and was expressed as a ratio or percentage of the control value in the Figures.

### Gelatin zymography analysis

MMP-9 expression and the activation of retinal homogenates and conditioned medium from cultured primary microglia were evaluated using gelatin zymography as previously described^53^. Clear zones (bands) in a blue background represent MMP-9 degradatory activity. The image was acquired using a IP-008-SP Photo-print digital imaging system (Vilber Lourmat, Marue La Vallee, France), and BIO-PROFIL Bio-1D light analytical software (Vilber Lourmat, Marue La Vallee, France) was used for the densitometric quantitation of the gelatinolytic zones.

### Statistical analyses

The experimental results were expressed as the mean ± SEM from the indicated number of experiments. The results were analyzed using one-way analysis of variance (ANOVA) with the Sigma Stat v3.5 software. The Student-Newman-Keuls test was used to evaluate statistically significant differences between groups. A *p*-value < 0.05 was considered statistically significant.

## Additional Information

**How to cite this article**: Lin, F.-L. *et al*. The natural retinoprotectant chrysophanol attenuated photoreceptor cell apoptosis in an *N*-methyl-*N*-nitrosourea-induced mouse model of retinal degeneration. *Sci. Rep.* **7**, 41086; doi: 10.1038/srep41086 (2017).

**Publisher's note:** Springer Nature remains neutral with regard to jurisdictional claims in published maps and institutional affiliations.

## Supplementary Material

Supplementary Information
