# Supplementary material for: The natural retinoprotectant chrysophanol attenuated photoreceptor cell apoptosis in an N-methyl-N-nitrosourea-induced mouse model of retinal degenaration
Source: Sci Rep. 2017 Jan 23;7:41086. doi: 10.1038/srep41086 (PMC5253624; doi:10.1038/srep41086)
Supplement: Supplementary Information [file srep41086-s1.pdf]

Supplementary Information

The natural retinoprotectant chrysophanol attenuated photoreceptor cell apoptosis in an *N*-methyl-*N*-nitrosourea-induced mouse model of retinal degenaration

Fan-Li Lin[1], Cheng-Hui Lin[2], Jau-Der Ho[3], Jing-Lun Yen[1], Hung-Ming Chang[4], George C.Y. Chiou[5], Yu-Wen Cheng[2,†], George Hsiao[1,*,†]

[1]Graduate Institute of Medical Sciences and Department of Pharmacology, School of Medicine, College of Medicine, Taipei Medical University, Taipei, Taiwan; [2]School of Pharmacy, College of Pharmacy, Taipei Medical University, Taipei, Taiwan; [3]Department of Ophthalmology, Taipei Medical University Hospital, Taipei, Taiwan [4]Department of Anatomy, School of Medicine, College of Medicine, Taipei Medical University, Taipei, Taiwan; [5]Department of Neuroscience and Experimental Therapeutics and Institute of Ocular Pharmacology, College of Medicine, Texas A&M Health Science Center, College Station, TX, USA. [*]Corresponding author. [†]The authors contributed equally to this work.

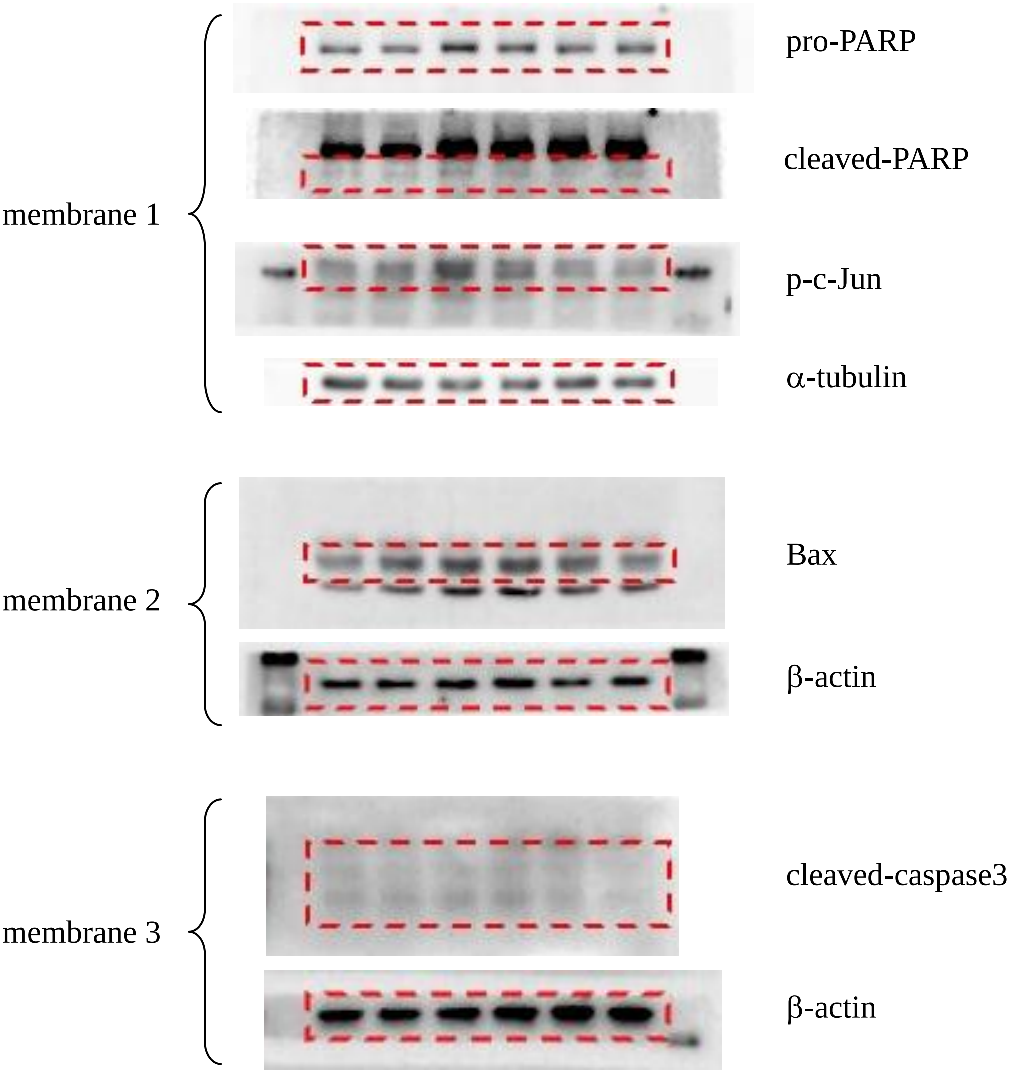

Supplementary Figure S1. Full-length western blot images of Figure 6.

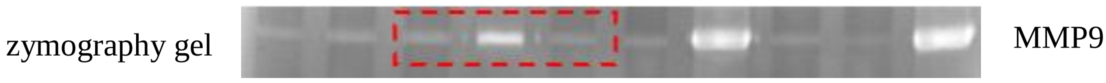

Supplementary Figure S2. Full-length zymography images of Figure 7.

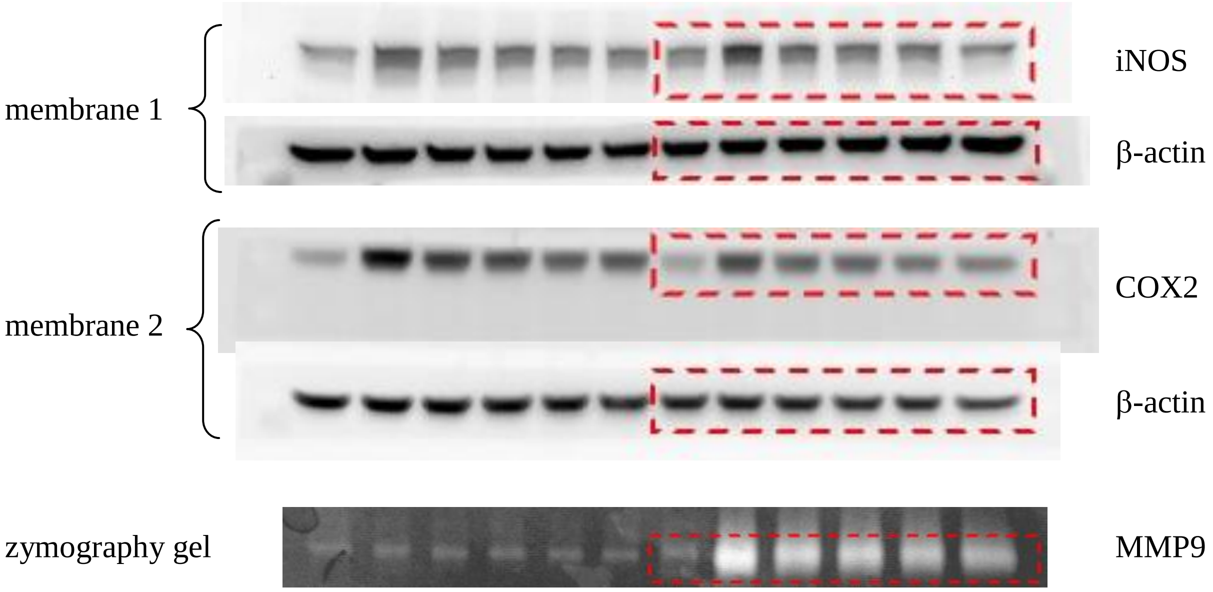

Supplementary Figure S3. Full-length western blot and zymography images of Figure 8.
